# Comment on Xiang et al. Association between the Triglyceride-Glucose Index and Vitamin D Status in Type 2 Diabetes Mellitus. *Nutrients* 2023, *15*, 639

**DOI:** 10.3390/nu15184068

**Published:** 2023-09-20

**Authors:** Roshan Kumar Mahat, Vedika Rathore

**Affiliations:** 1 Department of Biochemistry, Dharanidhar Medical College and Hospital, Keonjhar 758001, India; 2 Department of Biochemistry, Shyam Shah Medical College, Rewa 486001, India; ved_sin26@rediffmail.com

We read with great interest the article entitled “Association between the Triglyceride-Glucose Index and Vitamin D Status in Type 2 Diabetes Mellitus” by Xiang Q et al. [1] published in *Nutrients*. In this study, the authors aimed to investigate the association between the triglyceride-glucose index (TyG index) and vitamin D deficiency in individuals diagnosed with type 2 diabetes mellitus (T2DM). They found a negative association between the TyG index and 25-hydroxyvitamin D (25 (OH) D) and concluded that patients with a higher TyG index were more susceptible to an increased risk of vitamin D deficiency in T2DM. In addition, they calculated the cut-off point in the TyG index for predicting vitamin D deficiency in T2DM. Based on their findings, the cut-off point for detecting vitamin D deficiency in T2DM was determined to be 9.03, exhibiting a sensitivity of 75.0% and a specificity of 41.9%.

Xiang Q et al. [1] employed the following formula to calculate the TyG index: Ln [fasting triglyceride (mg/dL) × fasting glucose (mg/dL)/2]. While Xiang Q et al. [1] did not provide a specific reference for this formula, to the best of our knowledge, this formula was originally proposed by Simental et al. in 2008 [2]. However, Xiang Q et al. [1] have mentioned this formula in the Introduction section, citing different references [3,4]. Later in 2020, Simental et al. [5] clarified that there was a mistake in their first publication; they had conducted all the analyses and results using the following formula for the triglyceride and glucose (TyG) index instead: Ln [fasting triglycerides (mg/dL) × fasting glucose (mg/dL)]/2. Due to the slight disparity in these formulas, different TyG values were obtained. This discrepancy arises from the fact that the first formula divides the product of fasting triglyceride and glucose by 2 before taking the logarithm, whereas the second formula divides by 2 after the logarithmic operation is performed on the product of fasting triglyceride and glucose.

Therefore, Xiang et al. [1] committed an error during their calculation of the TyG index. While this mistake does not affect the predictive capability of the method, it does significantly alter the cut-off values. Therefore, we recommend that the authors, Xiang et al. [1], recompute their TyG index values using the following formula: Ln [fasting triglycerides (mg/dL) × fasting glucose (mg/dL)]/2. This will ensure that a more accurate cut-off value is obtained for the identification of vitamin D deficiency in individuals with type 2 diabetes mellitus.
